# The Variation in the Diastolic Period with Interventricular Septal Displacement and Its Relation to the Right Ventricular Function in Pulmonary Hypertension: A Preliminary Cardiac Magnetic Resonance Study

**DOI:** 10.3390/diagnostics12081970

**Published:** 2022-08-15

**Authors:** Fan Yang, Wen Ren, Dan Wang, Yan Yan, Yuan-Lin Deng, Zhen-Wen Yang, Tie-Lian Yu, Dong Li, Zhang Zhang

**Affiliations:** 1 Department of Radiology, Tianjin Medical University General Hospital, Tianjin 300052, China; 2 Department of Radiology, The Affiliated Suzhou Hospital of Nanjing Medical University, Suzhou 215008, China; 3 Department of Ultrasonography, Shanxi Bethune Hospital, Taiyuan 030032, China; 4 Department of Cardiology, Tianjin Medical University General Hospital, Tianjin 300052, China

**Keywords:** pulmonary hypertension, magnetic resonance imaging, cine, cardiovascular physiological phenomena, cardiac cycle, ventricular function

## Abstract

Background: Pulmonary hypertension (PH) is known to alter the biventricular shape and temporal phases of the cardiac cycle. The presence of interventricular septal (IVS) displacement has been associated with the severity of PH. There has been limited cardiac magnetic resonance (CMR) data regarding the temporal parameters of the cardiac cycle in PH. This study aimed to quantify the temporal changes in the cardiac cycle derived from CMR in PH patients with and without IVS displacement and sought to understand the mechanism of cardiac dysfunction in the cardiac cycle. Methods: Patients with PH who had CMR and right heart catheterization (RHC) examinations were included retrospectively. Patients were divided into an IVS non-displacement (IVS_ND_) group and an IVS displacement (IVS_D_) group according to IVS morphology, as observed on short-axis cine CMR images. Additionally, age-matched healthy volunteers were included as the health control (HC). Temporal parameters, IVS displacement, ventricular volume and functional parameters were obtained by CMR, and pulmonary hemodynamics were obtained by RHC. The risk stratification of the PH patients was also graded according to the guidelines. Results: A total of 70 subjects were included, consisting of 33 IVS_D_ patients, 15 IVS_ND_ patients, and 22 HC patients. In the IVS_ND_ group, only the right ventricle ejection fraction (RVEF) was decreased in the ventricular function, and no temporal change in the cardiac cycle was found. A prolonged isovolumetric relaxation time (IRT) and shortened filling time (FT) in both ventricles, along with biventricular dysfunction, were detected in the IVS_D_ group (*p* < 0.001). The IRT of the right ventricle (IRT_RV_) and FT of the right ventricle (FT_RV_) in the PH patients were associated with pulmonary vascular resistance, right cardiac index, and IVS curvature, and the IRT_RV_ was also associated with the RVEF in a multivariate regression analysis. A total of 90% of the PH patients in the IVS_D_ group were stratified into intermediate- and high-risk categories, and they showed a prolonged IRT_RV_ and a shortened FT_RV_. The IRT_RV_ was also the predictor of the major cardiovascular events. Conclusions: The temporal changes in the cardiac cycle were related to IVS displacement and mainly impacted the diastolic period of the two ventricles in the PH patients. The IRT and FT changes may provide useful pathophysiological information on the progression of PH.

## 1. Introduction

The right ventricle (RV) and left ventricle (LV) work within a distensible pericardium and are connected to each other through the interventricular septum (IVS), which shares myocardial fibers with both ventricles and accommodates the interactions between the two ventricles [1]. Therefore, the overloading of the RV pressure and volume affects not only the RV morphology and function, but also the LV morphology and function, in both the systolic and diastolic phases [2].

Increased pulmonary arterial pressure (PAP) can lead to prolonged and inhomogeneous RV contraction and has been associated with negative ventricular–ventricular interactions in pulmonary hypertension (PH) [3,4]. There is also evidence that the mechanism of RV-induced LV discoordination involves a combination of delayed early systolic electromechanical activation, late-systolic IVS shift, and prolonged post-systolic IVS thickening [5]. IVS displacement is defined as a flattening or bowing toward the LV [6], and it is observed in patients with PH. The presence of IVS displacement has been associated with the severity of PH [7] and may impair the LV filling dynamics [8]. IVS displacement is considered to be a consequence of the prolonged contraction of the RV free wall relative to that of the IVS and the LV free wall, causing interventricular relaxation dyssynchrony [9]. Therefore, IVS displacement causes the ventricular interdependency to become visible in PH. The simulations using the computational model have shown that the altered duration of the RV free wall contraction and profound IVS dyskinesia are associated with interventricular mechanical discoordination and decreased early LV filling in PH [10]. Nevertheless, the computational simulations of PH cannot explain the cases of all PH patients, especially in regard to the severe symptomatic PH patients.

Cardiac magnetic resonance (CMR) imaging is a non-invasive, robust diagnostic follow-up tool used for PH patients [11]. Cine CMR imaging is the reference standard for the evaluation of the morphology, volume, and function of both the LV and RV [12], and its value in the evaluation of patients with PH is increasingly recognized [12]. Cine CMR is able to describe the morphological changes in IVS to give a detailed assessment of the severity of PH [13]. It can display the opening and closure of the aortic valves, pulmonary arterial valves, mitral valves, and tricuspid valves distinctly [14], so that the temporal parameters of the cardiac cycle can be derived accurately. However, limited CMR data are available regarding the changes in the cardiac cycle in PH patients.

The goal of this study was to quantify the temporal changes in the cardiac cycle in PH patients with different IVS shapes and their relationship with the cardiac function using CMR, and to understand the mechanism of cardiac dysfunction in the cardiac cycle.

## 2. Materials and Methods

### 2.1. Study Design and Patient Enrollment

**Figure 1:**
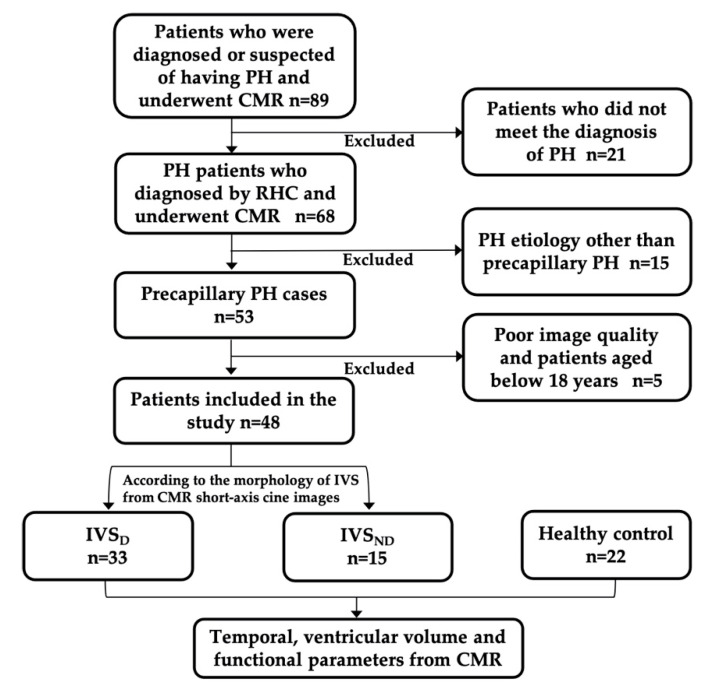
Patient flowchart. PH, pulmonary hypertension; CMR, cardiac magnetic resonance; RHC, right heart catheterization; IVS_ND_, pulmonary hypertension patients without interventricular septum displacement; IVS_D_, pulmonary hypertension patients with interventricular septum displacement.

Data were obtained from the records of adult patients who were diagnosed with or suspected of having PH from May 2012 to August 2018, who had been examined by CMR. The eligible patients retrospectively included in this study were those who were diagnosed with PH by right heart catheterization (RHC), as defined by the European Society of Cardiology [15], but who had not received any treatment. The interval between the CMR and the RHC examinations was less than a week. All the patients were in sinus rhythm. All patients whose etiology was not precapillary PH were excluded because of their different hemodynamic conditions. Precapillary PH was defined as a mean pulmonary arterial pressure (mPAP) of ≥25 mmHg with a pulmonary artery wedge pressure (PAWP) of ≤15 mmHg and pulmonary vascular resistance (PVR) of >3 Wood units, measured by RHC [15]. Patients who were aged below 18 years or those whose CMR image qualities did not meet the post-processing requirements were excluded. All the PH patients were divided into two groups according to IVS morphology, observed on short-axis cine CMR images. The PH patients with IVS flattening or even bowing toward the LV were classified as the IVS displacement (IVS_D_) group, while the PH patients without IVS displacement were classified as the IVS non-displacement (IVS_ND_) group. Additionally, 22 age-matched healthy volunteers with no evidence of any heart diseases also underwent CMR examination and were included as the health control (HC) group (Figure 1). The study was approved by the hospital research ethics committee and conducted in compliance with all the clinical practice requirements as prescribed by the committee. The requirement of informed consent was waived.

### 2.2. CMR Examination

CMR was performed on a 3.0 T MR scanner (GE Healthcare, Discovery MR 750, Milwaukee, WI, USA) with an 8-channel cardiac coil, using a vector-cardiographic method for electrocardiogram gating. The short-axis cine CMR images (slice thickness = 8 mm), 4-chamber cine images (slice thickness = 6 mm), and LV and RV outflow tracts (slice thickness = 6 mm) were acquired using fast imaging employing steady-state acquisition (FIESTA) during breath-holds. The acquisition parameters were as follows: 20 frames per cardiac cycle, repetition time 3.40 to 3.60 ms, echo time 1.50 to 1.60 ms, flip angle = 45°, bandwidth = 125 KHz/pixel, field of view = 35 cm × 35 cm, matrix = 224 × 224, and NEX = 1.

### 2.3. CMR Image Analysis

The CMR image analysis was performed with Report Card 3.7 on GE Advantage Workstation 4.6. The analysis of the IVS morphology was performed using the short-axis cine CMR images, as previously described in the literature [12,13,14], including the interventricular septal curvature (C_IVS_) and curvature ratio (CR). The RV and LV function analysis was also performed using the short-axis cine CMR images. The RV and LV endocardial and epicardial borders were automatically traced with manual adjustments to obtain the end-diastolic volume (EDV), end-systolic volume (ESV), stroke volume (SV) and ejection fraction (EF). The papillary muscles and trabeculae were included in the ventricular cavity volume. The myocardial mass (MM) was calculated by multiplying the volume of the ventricular myocardium in the end-diastolic phase with its density (1.05 g/cm^3^). The ventricular mass index (VMI) was calculated by dividing the RVMM by the LVMM. All the cardiac functional parameters were divided by the specific body surface area (BSA) for normalization and recorded as the end-diastolic volume index (EDVI), end-systolic volume index (ESVI), stroke volume index (SVI) and myocardial mass index (MMI), respectively.

The opening and closure of the aortic valves, pulmonary valves, mitral valves and tricuspid valves were reviewed using the cine CMR images. The aortic valves and pulmonary valves were reviewed using the outflow tract cine images of the LV and RV, respectively. The mitral valves and tricuspid valves were observed using the 4-chamber cine view. The opening time and closure time of the valves were normalized and recorded as the percentage of the R-R interval. The cardiac cycle was composed of an isovolumetric relaxation phase, a filling phase, an isovolumetric contraction phase and an ejection phase. The durations of the four phases were calculated as follows (isovolumetric relaxation time, IRT; filling time, FT; isovolumetric contraction time, ICT; ejection time, ET; tricuspid valve, T; pulmonary artery valve, P; mitral valve, M; aortic valve, A; open, o; closure, c):

IRT_RV_ = T_O_ − P_C_

FT_RV_ = T_C_ − T_O_

ICT_RV_ = P_O_ − T_C_

ET_RV_ = P_C_ − P_O_

IRT_LV_ = M_O_ − A_C_

FT_LV_ = M_C_ − M_O_

ICT_LV_ = A_O_ − M_C_

ET_LV_ = A_C_ − A_O_

To assess the inter-observer and intra-observer reproducibility of the temporal parameters of the cardiac cycle, 18 (26%) PH patients were selected randomly and re-examined independently by the two readers (FY, 6 years CMR experience and DW, 3 years CMR experience). For the intra-observer reproducibility, one observer (FY) repeated the measurements four weeks later. The intra-class correlation coefficient was used to assess the reproducibility.

### 2.4. Risk Stratification, Follow-Up, and Study Endpoint

In line with the risk assessment instrument from the abbreviated version of the 2015 European Society of Cardiology (ESC)/European Respiratory Society (ERS) risk stratification strategy [15], all the PH patients were graded according to the World Health Organization functional classification (WHO FC), including 6 min walking distance (6 MWD), brain natriuretic peptide (BNP), N-terminal prohormone of the brain natriuretic peptide (NT-proBNP), mean right atrial pressure (mRAP), cardiac index (CI) and mixed venous oxygen saturation (SvO2). For each patient, the sum of all grades was divided by the number of available variables and rounded to the next integer to define the risk group. The cut-off values proposed in the guidelines were graded from 1–3 (1: low risk, 2: intermediate risk, 3: high risk). The overall treatment goal for patients with PH is to achieve a low-risk status; thus, the PH patients were divided into the low-risk group and the intermediate- and high-risk groups.

All the PH patients were followed up with a census date of 12 January 2021. The designed primary endpoint was defined as major cardiovascular events (MACE), which included hospitalization for heart failure, lung transplantation, malignant ventricular arrhythmia and death.

### 2.5. Statistical Analyses

SPSS 23.0 was used for all the statistical analyses. All the continuous variables were presented as medians and interquartile ranges (IQR) when the variables were not normally distributed. The Kruskal–Wallis one-way ANOVA test, with Bonferroni correction post hoc analysis, was used to compare the continuous variables among the three groups of HC, IVS_ND_ and IVS_D_. The categorical variables were presented as frequencies (%) and compared using Fisher’s exact test. The correlation between the parameters was calculated with Spearman’s correlation coefficient. Multivariable stepwise regression analysis was used to explore the factors associated with RVEF. Each variable with a significant association (*p* < 0.05) in the univariate analysis was introduced into the regression model. The differences in the ventricular function and the temporal parameters between the low-risk group and the intermediate- and high-risk groups were tested by the Mann–Whitney U test. The univariable and multivariable Cox regression models included demographic factors, clinical factors, laboratory tests, and RHC and CMR variables (variables whose *p*-value was <0.15 in the univariable Cox regression analysis were included in the multivariable Cox regression analysis). All the analyses were two-sided, and *p*-values of <0.05 were considered to be statistically significant.

## 3. Results

### 3.1. Patient Characteristics

The clinical characteristics of the subjects are presented in Table 1. A total of 48 consecutive precapillary PH patients confirmed by RHC were enrolled, including 29 patients with PAH, 18 patients with chronic thromboembolic pulmonary hypertension (CTEPH), and one patient who had PH with unclear and/or multifactorial mechanisms. There were no significant differences in age, sex or BSA among the IVS_ND_ group, the IVS_D_ group and the HC group. The IVS_D_ group had significantly decreased 6MWD and increased NT-proBNP and WHO FC levels. The HR was significantly faster in the IVS_D_ group than in the IVS_ND_ group (*p* < 0.05) and in the HC group (*p* < 0.001). No significant differences between the IVS_ND_ group and the IVS_D_ group in the proportion of PH subsets (*p* > 0.05) were observed. The mPAP, PVR, CI and mRAP were higher in the IVS_D_ group than in the IVS_ND_ group (*p* < 0.05).

### 3.2. IVS Morphology and Cardiac Functional Parameters

The IVS_D_ group had significantly decreased C_IVS_ and CR when compared with the IVS_ND_ group and the HC group (Table 2).

**Figure 2:**
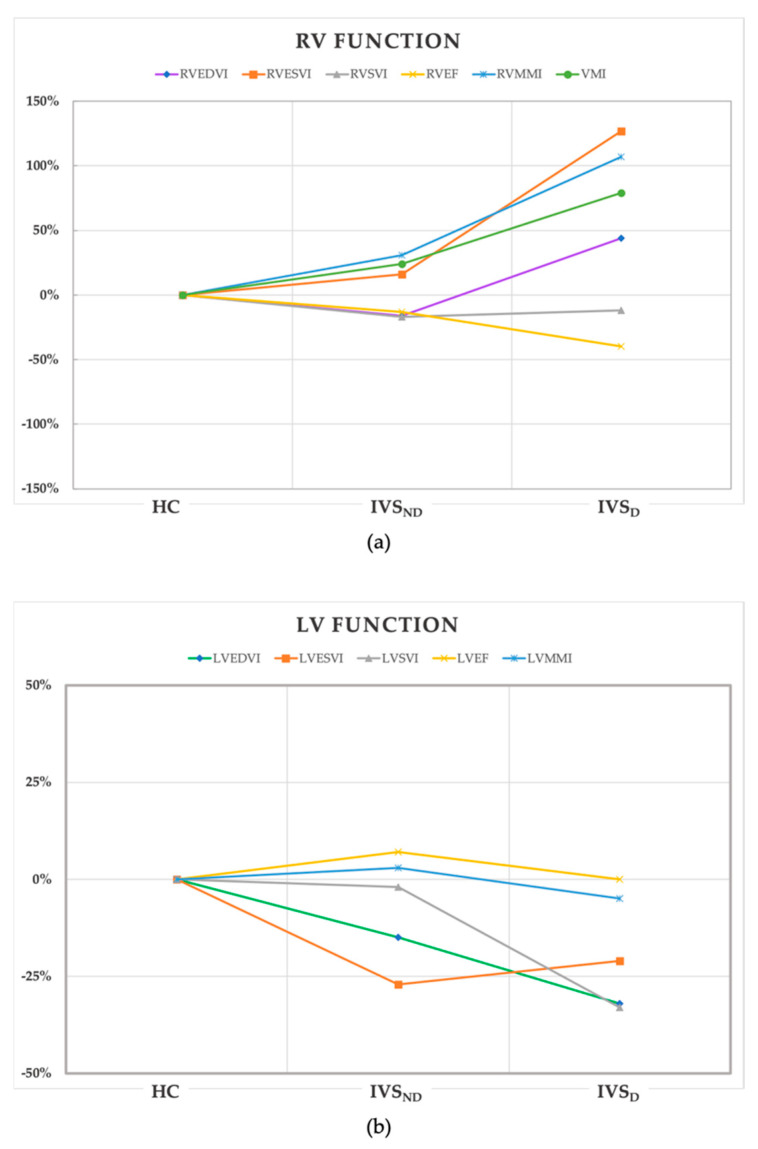
The trend of the percentage changes in the RV (**a**) and LV (**b**) function from the HC to IVS_D_ group. All of the values of the coordinate points were calculated from the percentage change of the median of the parameters in the IVS_ND_ and IVS_D_ groups using the HC as the baseline. HC, health control; IVS_ND_, pulmonary hypertension patients without interventricular septum displacement; IVS_D_, pulmonary hypertension patients with interventricular septum displacement; RV, right ventricle; LV, left ventricle; EDVI, end-diastolic volume index; ESVI, end-systolic volume index; SVI, stroke volume index; EF, ejection fraction; MMI, myocardial mass index; VMI, ventricular mass index.

Figure 2 shows the trend of the percentage changes in the RV (a) and LV (b) function from the HC to the IVS_D_ group. The detailed differences in the above functional parameters among the groups are shown in the Figure S1. For the RV parameters (Figure 2a), the IVS_D_ patients had increased RVEDVI and RVESVI, and decreased RVEF when compared with the IVS_ND_ group and the HC (*p* < 0.01). The RVEF was the only decreased parameter in the IVS_ND_ group compared with the HC (*p* < 0.05). While there was no significant difference in the RVSVI among the three groups (*p* > 0.05), among the LV parameters (Figure 2b), the LVEDVI, LVESVI, and LVSVI were decreased in the IVS_D_ patients (*p* < 0.001). There was no significant difference in the LVEF among the three groups (*p* > 0.05) (Table 2). The RVMMI and VMI of the IVS_D_ group were increased (*p* < 0.01), but the LVMMI showed no difference.

### 3.3. Temporal Parameters in the Cardiac Cycle

**Figure 3:**
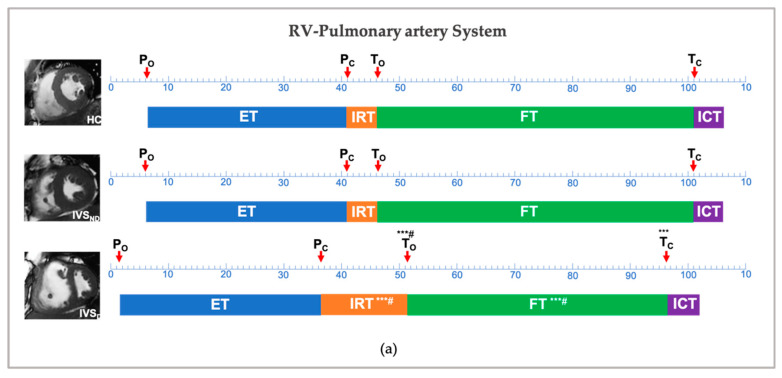

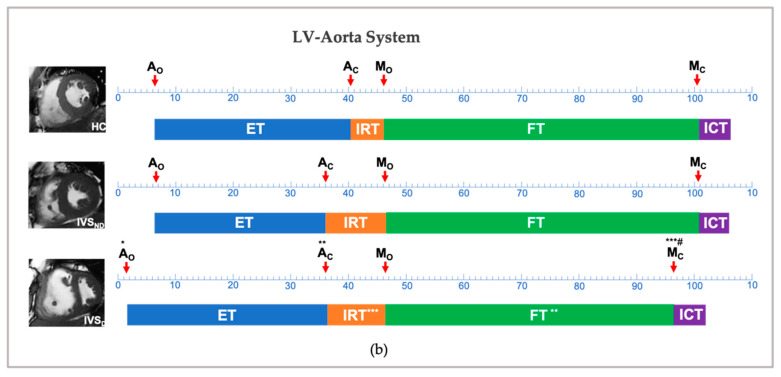
Temporal changes between the HC, IVS_ND_ and IVS_D_ in the right ventricle-pulmonary artery system (**a**) and left ventricle-aorta system (**b**). * *p* < 0.05, ** *p* < 0.01, *** *p* < 0.001: versus HC; ^#^ *p* < 0.05: versus IVS_ND_; P, pulmonary artery valve; A, aortic valve; M, mitral valve; T, tricuspid valve; ICT, isovolumetric contraction time; IRT, isovolumetric relaxation time; ET, ejection time; FT, filling time; HC, health control; IVS_ND_, pulmonary hypertension patients without interventricular septum displacement; IVS_D_, pulmonary hypertension patients with interventricular septum displacement.

Table 3 and Figure 3 present the characteristics of the temporal parameters in the cardiac cycle. All the temporal parameters showed no difference between the IVS_ND_ and HC groups.

Compared with the HC group, the IVS_D_ showed no difference in the P_O_, P_C_, ET_RV_ and ICT_RV_ in terms of the RV-pulmonary artery system, while this group showed a delayed T_O_, an advanced T_C_, a longer IRT_RV_ and a shorter FT_RV_ (*p* < 0.001). With regards to the LV-aorta system, although both the A_O_ and the A_C_ were advanced, the ET_LV_ and the ICT_LV_ showed no differences in the IVS_D_ group. The IVS_D_ group showed no differences in the M_O_, while showing an advanced M_C_, a longer IRT_LV_ and a shorter FT_LV_ (*p* < 0.001).

Comparing the two groups of IVS_D_ and IVS_ND_, a longer IRT_RV_ and shorter FT_RV_ were detected along with a delayed T_O_ in the IVS_D_ group (*p* < 0.05) (Table 3). However, in the temporal parameters of the LV, no differences were detected, except an advanced M_C_ (*p* < 0.05).

The intra- and inter-observer reproducibility results using intra-class correlation coefficients are shown in the Supplementary Materials, Table S1. The inter-observer and intra-observer variability results for all the temporal parameters were very low.

### 3.4. Correlation of the Temporal Parameters with the RHC and CMR Data

The IRT_RV_ was significantly correlated with the PVR, CI and C_IVS_ (r = 0.38, r = −0.34, r = −0.49, *p* < 0.05). The FT_RV_ was significantly correlated with the mPAP, PVR, CI and C_IVS_ (r = −0.46, r = −0.52, r = 0.47, r = 0.55, *p* < 0.01). There were significant correlations between some ventricular functional parameters and mPAP, PVR, CI and C_IVS_, respectively (*p* < 0.05) (Table 4).

The multivariate linear regression included age, sex, 6MWD, NT-proBNP, and RHC and CMR variables and showed that the IRT_RV_, LVEF and PVR were significantly associated with the RVEF (Table 5).

### 3.5. Differences in the CMR and RHC Characteristics Based on Risk Stratification

Table 1 shows that 40% of the IVS_ND_ group and 90% of the IVS_D_ group were in the intermediate- and high-risk categories. Patients in the intermediate- and high-risk groups had a significantly increased RVEDVI, RVESVI and RVMASSI VMI, and a lower RVEF, LVEDVI, LVSVI and CR compared to the low-risk group (*p* < 0.05) (Table 6). Patients in the intermediate- and high-risk groups displayed a delayed T_O_, a longer IRT_RV_ and a shortened FT_RV_ (*p* < 0.01).

### 3.6. Survival Analysis

Twelve patients (25%) with PH died during the median follow-up period of 62 months (interquartile range: 38–67 months). The univariate Cox proportional hazards regression analysis of all the PH patients showed that age, WHO FC, 6MWD, IRT_RV_, CR and mPAP were associated with the MACE. The multivariable analysis showed that the IRT_RV_ and mPAP were the significant predictors of the MACE (Table 7).

## 4. Discussion

This cardiac MR study has provided detailed information about the temporal variations in the cardiac cycle in the progression of PH patients. The main variations were in the diastolic period, and the PH patients with IVS displacement had a longer IRT and shorter FT for both the RV and LV, and a prolonged IRT and shortened FT of the RV were associated with the RV afterload. Additionally, the IRT_RV_ may have the potential to predict the prognosis for PH patients. However, the PH patients without IVS displacement showed no temporal variations in the cardiac cycle.

Increased PVR and elevated PAP are the main factors responsible for PH, leading to vascular remodeling and cardiac remodeling [16]. During the compensation stage, the PH patients showed a mildly decreased RV contractile function and sustained LV function. The filling pressure and interventricular pressure gradients were still normal [10,17]. Previous studies showed that RV contractile dysfunction was significantly associated with the severity of PH and the curvature of the IVS showed a strong correlation with the sPAP [6,18]. The current findings showed that the only impaired cardiac functional parameter was the RVEF in the PH patients without IVS displacement.

Although increased mPAP and PVR reflect the severity of PH, increased mPAP and PVR do not reflect an adverse ventricular–ventricular interaction, interventricular pressure gradient and interventricular septal motion directly [10]. The increased afterload and impaired ventricular interaction deteriorate both ventricles to the decompensation state. The RV is remodeled both concentrically and eccentrically with the RV dilatation and myocardial hypertrophy [19]. The IVS becomes flattened, and even bows to the LV. As the results of this study showed, the C_IVS_ and CR were both reduced in the IVS_D_ patients. The RVEDVI, RVESVI and RVMMI were significantly increased and the RVEF was further decreased in the IVS_D_ patients compared with those of the IVS_ND_ patients and HC patients. The LVEDVI, LVESVI and LVSVI were decreased with the IVS displacement and LV compression.

Furthermore, RV pressure loading influences not only the IVS morphology but also the temporal phases of the cardiac cycle [2]. In general, the opening and closure of the atrioventricular valves are mainly dependent on the different pressure gradients between the atrium and ventricle. When the intraventricular pressure drops below the intra-atrial pressure, the atrioventricular valve opens, while the atrioventricular valve closes when the intraventricular pressure rises above the intra-atrial pressure [14].

With respect to the pulmonary artery-RV system, our study identified delayed To, advanced Tc, prolonged IRT_RV_ and shortened FT_RV_ in the IVS_D_ patients. In PH patients with IVS displacement, the increased RV pressure and inefficient RV diastolic function could result in a prolonged IRT_RV_, which could in turn decrease the intraventricular pressure. Moreover, increased RV pressure would promote the closure of the tricuspid valve and shorten the FT_RV_ accordingly. An increased afterload would reduce the myocyte velocity and prolong the myocyte shortening. Thus, RV actin-myosin cross-bridge cycling may result in a stiffer myocardium near the end of the ventricular ejection, prolonging the IRT and hampering the early relaxation [20]. The IRT_RV_ was prolonged in PH patients when compared with the controls, resulting in a noticeable delay in atrioventricular opening [21]. The shortened FT_RV_ then impedes its filling, which has an essential impact on the RV systolic function [2].

With respect to the LV-aorta system, the IVS_D_ patients displayed advanced Mc, prolonged IRT_LV_ and shortened FT_LV_. This was linked to IVS displacement. The increased RV pressure causing the RV dilatation and IVS flattening or even bowing to the LV would result in a smaller LV cavity, which leads to the LV underfilling and reduction in the LVSV. RV dilatation and IVS displacement had a further deleterious effect on ventricular interactions [4]. Under these circumstances, a prolonged IRT and shortened FT of the LV and RV could cause ventricular underfilling and decrease the systolic function of both ventricles [10,19,21,22,23].

Our findings also showed that IRT_RV_, FT_RV_ and some of the cardiac functional parameters (such as RVEDVI, RVESVI, RVEF and VMI) were associated with PVR, CI and C_IVS_. Additionally, the IRT_RV_, LVEF and PVR were associated with the RVEF in the multivariate regression analysis. All these findings indicate that the IVS displacement and increased RV afterload were not the only factors related to ventricular dysfunction. The temporal variations of both the IRT_RV_ and FT_RV_ in the cardiac cycle may also be associated with an IVS morphological abnormality and impaired ventricular function, especially in the case of the IRT_RV_.

Guidelines on the diagnosis and treatment of PH, published by the European Society of Cardiology and European Respiratory Society, state that the overall treatment goal for patients with PH is to achieve a low-risk status, which is usually associated with good exercise capacity, good quality of life, good RV function and a low-mortality risk [15]. The main indicators for the risk stratification in PH are WHO FC, 6MWD, NT-proBNP/BNP and CI [11], and the corresponding results of this study were consistent with the guidelines. Most of the PH patients with IVS displacement were stratified into the intermediate- and high-risk categories, reaching as high as 90% in this study, and they showed delayed To, prolonged IRT_RV_ and shortened FT_RV_. These findings indicate that the temporal variations of the IRT_RV_ and FT_RV_ in cardiac cycle could also inform the risk stratification of PH patients. Moreover, the multivariable Cox proportional hazards regression analysis showed that the IRT_RV_ was the significant predictor of the MACE. This indicates that the IRT_RV_ might serve as a potential prognostic factor for PH patients.

By applying the current methods in the clinics, we can not only evaluate the function and morphology of the ventricles, but also provide extra temporal information for PH patients, without introducing additional CMR sequences and scanning times. This pathophysiological information may give the doctors a profound understanding of both the progression of PH and the interaction between the ventricles, offering help in the guidance of the treatment strategy for individuals with PH. IVS displacement, prolonged IRT_RV_, and shortened FT_RV_ indicate RV failure in patients with precapillary-PH, and, more seriously, predict clinical worsening [24]. On the contrary, the reversible displacement of IVS is useful in predicting the alleviation of PH [25]. The temporal parameters of the diastole may have prognostic value and may act as indicators of effective treatment. Cardiac resynchronization therapy is a potential strategy for patients with severe symptomatic PH [26]. The findings regarding the changes in the temporal parameters might provide additional implications for intervention. Large and long-term prospective studies on IVS displacement and the associated temporal changes are needed to confirm this.

This study has several limitations. Firstly, this is a single-center study with a small sample size. The current results and discoveries should be further validated using a large PH population. Next, although the four phases in the cardiac cycle were clearly set out in this study, the pilot temporal parameter method, which used CMR cine data with images including 20 frames from multiple cardiac cycles, might have caused sampling errors or inaccuracies. All the patients were in sinus rhythm, and this could minimize the sampling errors. Finally, the heterogeneous etiology causing precapillary-PH might have led to other uncontrolled factors in the analysis. Further exploration is needed.

With the progression of PH, the diastolic period variations in the RV and LV were related to IVS displacement and ventricular dysfunction. The temporal variations in the cardiac cycle may also be possible parameters, which can explain the severity of the disease and provide useful information about the pathophysiological mechanism of the ventricular dysfunction and indicate the prognosis for PH patients.

## Figures and Tables

**Table 1:** Demographic, clinical and RHC characteristics in the health control and PH patients.

	HC (*n* = 22)	IVS_ND_ (*n* = 15)	IVS_D_ (*n* = 33)
**Demographics**			
Female, *n* (%)	20 (90.9)	14 (93.3)	27 (81.8)
Age, years	43 (35–49)	52 (37–62)	40 (32–62)
PAH/CTEPH	--	8/21	7/11
WHO FC I/II/III/IV	--	0/14/1/0	0/13/17/3 ^###^
6MWD, m	--	444 (302–463)	235 (157–339) ^###^
NT-proBNP, pg/mL	--	97 (56–245)	1678 (634–1961) ^###^
HR, bpm	68 (63–73)	71 (66–79)	84 (78–93) *** ^#^
BSA, m^2^	1.65 (1.58–1.77)	1.66 (1.51–1.75)	1.69 (1.50–1.76)
Low-risk/intermediate and high risk	--	9/6	3/30
**RHC measurements**			
mPAP, mmHg	--	39 (33–46)	54 (41–62) ^##^
PVR, Wood	--	10 (7–14)	16 (11–21) ^##^
CI, L/min/m^2^	--	2.3 (2.1–3.1)	1.9 (1.5–2.5) ^#^
mRAP, mmHg	--	5 (3–6)	6 (5–9) ^#^
PAWP, mmHg	--	10 (7–12)	9 (6–10)

*** *p* < 0.001: versus HC; ^#^ *p* < 0.05, ^##^ *p* < 0.01, ^###^ *p* < 0.001: versus IVS_ND._ HC, health control; IVS_ND_, pulmonary hypertension patient without interventricular septum displacement; IVS_D_, pulmonary hypertension patient with interventricular septum displacement; HR, heart rate; BSA, body surface area; WHO FC, World Health Organization functional classification; 6MWD, 6 min walking distance; NT-proBNP, N-terminal prohormone of the brain natriuretic peptide; PAH, pulmonary arterial hypertension; CTEPH, chronic thromboembolic pulmonary hypertension; RHC, right heart catheterization; mPAP, mean pulmonary arterial pressure; PVR, pulmonary vascular resistance; CI, cardiac index; mRAP, mean right atrium pressure; PAWP, pulmonary arterial wedge pressure.

**Table 2:** CMR-derived morphologic and functional parameter characteristics in the health control and PH patients.

	HC (*n* = 22)	IVS_ND_ (*n* = 15)	IVS_D_ (*n* = 33)
RVEDVI, mL/m^2^	81 (71–87)	68 (61–93)	117 (104–140) *** ^###^
RVESVI, mL/m^2^	37 (32–43)	43 (36–49)	84 (66–95) *** ^###^
RVSVI, mL/m^2^	42 (38–49)	35 (27–42)	37 (29–47)
RVEF, %	52 (50–60)	45 (38–50) *	31 (25–40) *** ^##^
RVMMI, g/m^2^	13 (10–17)	17 (13–19)	27 (20–31) *** ^##^
LVEDVI, mL/m^2^	80 (72–90)	68 (53–78)	54 (42–48) ***
LVESVI, mL/m^2^	33 (29–41)	24 (21–35)	26 (17–29) ***
LVSVI, mL/m^2^	45 (42–52)	44 (32–50)	30 (23–39) *** ^#^
LVEF, %	57 (52–67)	61 (58–64)	57 (53–61)
LVMMI, g/m^2^	40 (37–48)	41 (36–47)	42 (36–48)
VMI	0.34 (0.27–0.42)	0.42 (0.30–0.46)	0.61 (0.48–0.76) *** ^##^
C_IVS_	0.07 (0.06–0.07)	0.06 (0.04–0.07)	0.01 (−0.01–0.02) *** ^###^
CR	1.00 (0.95–1.04)	0.88 (0.71–0.92)	0.24 (−0.23–0.45) *** ^###^

* *p* < 0.05, *** *p* < 0.001: versus HC; ^#^ *p* < 0.05, ^##^ *p* < 0.01, ^###^ *p* < 0.001: versus IVS_ND_; CMR, cardiac magnetic resonance; HC, health control; IVS_ND_, pulmonary hypertension patient without interventricular septum displacement; IVS_D_, pulmonary hypertension patient with interventricular septum displacement; RV, right ventricle; LV, left ventricle; EDVI, end-diastolic volume index; ESVI, end-systolic volume index; SVI, stroke volume index; EF, ejection fraction; MMI, myocardial mass index; VMI, ventricular mass index; C_IVS_, interventricular septal curvature; CR, curvature ratio.

**Table 3:** CMR-derived temporal parameters of the three groups.

Temporal Parameters	HC (*n* = 22)	IVS_ND_ (*n* = 15)	IVS_D_ (*n* = 33)
P_O_, %	6.1 (5.6–6.2)	6.0 (1.2–10.8)	1.3 (−3.4–6.2)
P_C_, %	41.0 (36.2–41.3)	40.8 (36.1–41.3)	36.3 (33.2–38.9)
A_O_, %	6.1 (4.8–6.2)	6.0 (1.1–10.7)	1.2 (−3.5–6.2) *
A_C_, %	41.0 (36.1–41.2)	36.1 (36.0–41.0)	36.1 (31.5–36.3) **
M_O_, %	46.1 (41.1–51.0)	46.2 (41.3–51.5)	46.4 (43.8–48.4)
M_C_, %	101.0 (96.2–101.1)	101.0 (91.5–101.1)	91.6 (86.7–96.3) *** ^#^
T_O_, %	46.1 (41.1–51.0)	46.2 (45.9–51.2)	51.3 (46.5–54.0) *** ^#^
T_C_, %	101.1 (99.7–101.1)	101.0 (91.5–101.1)	96.2 (91.5–96.4) ***
ICT_RV_, %	5.0 (4.9–5.3)	5.00 (4.9–10.0)	5.0 (4.9–10.0)
ET_RV_, %	35.0 (32.4–38.1)	30.0 (30.0–40.0)	35.0 (30.1–40.0)
IRT_RV_, %	5.0 (4.9–5.3)	9.9 (5.1–15.0)	15.0 (10.1–19.9) *** ^#^
FT_RV_, %	55.0 (50.0–56.3)	50.0 (45.0–55.0)	44.2 (35.7–49.9) *** ^#^
ICT_LV_, %	5.0 (5.0–10.0)	5.0 (4.9–10.0)	9.9 (5.0–10.1)
ET_LV_, %	35.0 (30.0–35.6)	30.0 (30.0–35.0)	35.0 (30.0–35.1)
IRT_LV_, %	5.1 (5.0–10.0)	9.9 (5.1–15.0)	13.4 (10.0–15.0) ***
FT_LV_, %	52.5 (49.9–55.1)	50.1 (39.9–60.0)	45.1 (40.1–50.0) **

* *p* < 0.05, ** *p* < 0.01, *** *p* < 0.001: versus HC; ^#^ *p* < 0.05: versus IVS_ND_; HC, health control; IVS_ND_, pulmonary hypertension patient without interventricular septum displacement; IVS_D_, pulmonary hypertension patient with interventricular septum displacement; RV, right ventricle; LV, left ventricle; P, pulmonary artery valve; A, aortic valve; M, mitral valve; T, tricuspid valve; ICT, isovolumetric contraction time; IRT, isovolumetric relaxation time; ET, ejection time; FT, filling time.

**Table 4:** Spearman correlations *r* between the CMR data and RHC data.

	mPAP	PVR	CI	Civs
RVEDVI	0.19	0.36 *	−0.35 *	−0.72 ***
RVESVI	0.34 *	0.53 **	−0.50 ***	−0.79 ***
RVEF	−0.50 **	−0.56 **	0.52 ***	0.67 ***
RVMMI	0.25	0.43 **	−0.41 **	−0.67 **
LVEDVI	−0.50 **	−0.41 **	0.40 **	0.44 ***
LVESVI	−0.39 **	−0.32 *	0.35 *	0.29 ***
LVSVI	−0.51 **	−0.43 **	0.39 **	0.48 *
VMI	0.41 **	0.46 **	−0.44 **	−0.66 ***
IRT_RV_	0.27	0.38 **	−0.34 *	−0.49 ***
FT_RV_	−0.46 **	−0.52 ***	0.47 **	0.55 ***

* *p* < 0.05, ** *p* < 0.01, *** *p* < 0.001. CMR, cardiac magnetic resonance; RHC, right heart catheterization; RV, right ventricle; LV, left ventricle; EDVI, end-diastolic volume index; ESVI, end-systolic volume index; SVI, stroke volume index; EF, ejection fraction; MMI, myocardial mass index; VMI, ventricular mass index; mPAP, mean pulmonary arterial pressure; PVR, pulmonary vascular resistance; CI, cardiac index; C_IVS_, interventricular septal curvature. IRT, isovolumetric relaxation time; FT, filling time.

**Table 5:** Multiple linear regression analysis for RVEF.

Variates	Unstandardized Coefficients		Standardized Coefficients	*t*	*p*-Value
	B (95% CI)	Std. Error	Beta		
IRT_RV_	−0.456 (−0.990–−0.246)	0.188	−0.293	−2.426	0.019
LVEF	0.490 (0.128–0.852)	0.180	0.308	2.728	0.009
PVR	−0.618 (−0.835–−0.077)	0.185	−0.403	−3.350	0.002

The correlation coefficient between the result of the model and the CMR-derived RVEF was as follow, R = 0.667, R^2^ = 0.444, adjusted R^2^ = 407, F = 11.733, *p* < 0.001. RVEF, right ventricular ejection fraction; LVEF, left ventricular ejection fraction; RV, right ventricle; IRT, isovolumetric relaxation time.

**Table 6:** Comparison of the CMR-derived indices and RHC characteristics of the PH patients based on risk stratification.

Variates	Low Risk (*n* = 12)	Intermediate and High Risk (*n* = 36)	*p*-Value
RVEDVI, mL/m^2^	89 (66–93)	112 (91–135)	0.008 *
RVESVI, mL/m^2^	47 (36–53)	80 (57–94)	0.001 *
RVEF, %	46 (40–50)	32 (27–40)	<0.001 *
RVMASSI, g/m^2^	17 (14–23)	23 (19–30)	0.012 *
LVEDVI, mL/m^2^	70 (56–81)	55 (43–70)	0.034 *
LVSVI, mL/m^2^	41 (31–53)	32 (23–41)	0.024 *
VMI	0.45 (0.32–0.50)	0.60 (0.44–0.72)	0.022 *
CR	0.78 (0.34–0.91)	0.29 (−0.21–0.69)	0.017 *
T_O_, %	46.2 (42.5–51.1)	51.3 (46.4–55.2)	0.005 *
IRT_RV_, %	9.9 (5.1–13.8)	15.0 (10.0–19.7)	0.009 *
FT_RV_, %	50.0 (49.9–55.0)	43.6 (35.0–49.9)	0.001 *

* *p* < 0.05.

**Table 7:** Univariable and multivariable Cox proportional hazard analysis for the MACE.

Variates	Univariate		Multivariate	
	HR (95% CI)	*p*-Value	HR (95% CI)	*p*-Value
age	0.971 (0.940–1.004)	0.08		
WHO FC	2.227 (1.039–4.774)	0.04		
6MWD	0.996 (0.991–1.000)	0.05		
IRT_RV_	0.946 (0.881–1.017)	0.13	0.930 (0.867–0.997)	0.04
CR	0.421 (0.152–1.164)	0.10		
mPAP	1.037 (1.004–1.072)	0.03	1.046 (1.012–1.081)	0.007

MACE, major cardiovascular event; HR, hazard ratio; CI, confidence interval.

## Data Availability

Data available on request.

## Supplementary Materials

The following supporting information can be downloaded at: https://www.mdpi.com/article/10.3390/diagnostics12081970/s1, Figure S1: The differences in RV and LV function among the HC, IVS_ND_, and IVS_D_ groups; Table S1: Reproducibility using intra-class correlation coefficients of temporal parameters.
