# Supplementary material for: The Variation in the Diastolic Period with Interventricular Septal Displacement and Its Relation to the Right Ventricular Function in Pulmonary Hypertension: A Preliminary Cardiac Magnetic Resonance Study
Source: Diagnostics (Basel). 2022 Aug 15;12(8):1970. doi: 10.3390/diagnostics12081970 (PMC9407232; doi:10.3390/diagnostics12081970)
Supplement: Supplementary file 1 [file diagnostics-12-01970-s001.zip › diagnostics-1818355-supplementary.pdf]

Figure S1. The differences in RV and LV function among the HC, IVS_{ND}, and IVS_{D} groups.

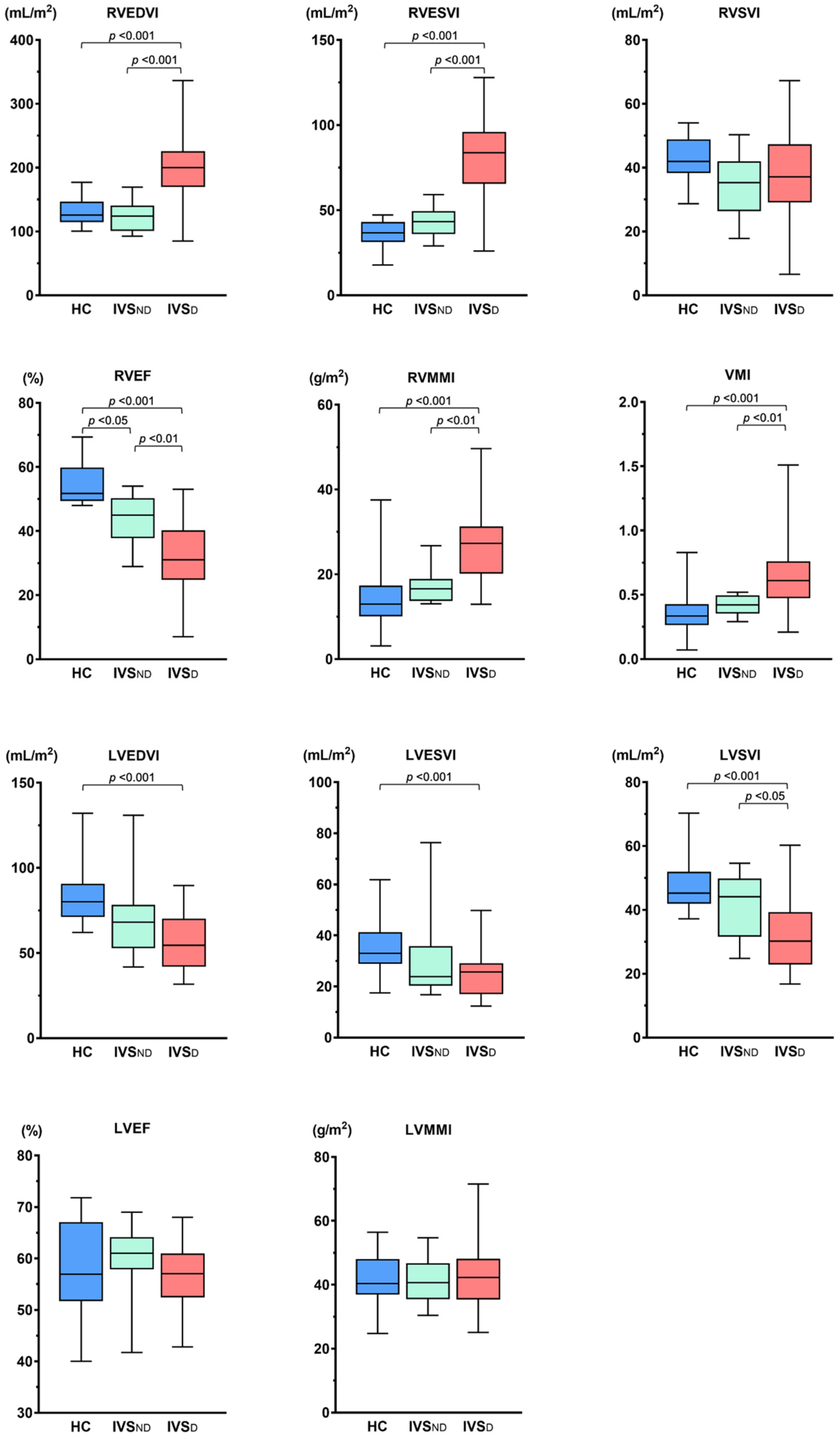

HC, health control; IVS_{ND}, pulmonary hypertension patient without interventricular septum

displacement; IVS_D, pulmonary hypertension patient with interventricular septum displacement; RV, right ventricle; LV, left ventricle; EDVI, end-diastolic volume index; ESVI, end-systolic volume index; SVI, stroke volume index; EF, ejection fraction; MMI, myocardial mass index; VMI, ventricular mass index.

Table S1. Reproducibility using intra-class correlation coefficients of temporal parameters.

	Intra-observer	Inter-observer
P_O, %	0.957	0.965
P_C, %	0.956	0.955
A_O, %	0.883	0.954
A_C, %	0.859	0.973
M_O, %	0.925	0.945
M_C, %	0.870	0.958
T_O, %	0.935	0.942
T_C, %	0.870	0.921
ICT_{RV}, %	0.734	0.854
ET_{RV}, %	0.922	0.940
IRT_{RV}, %	0.887	0.915
FT_{RV}, %	0.922	0.940
ICT_{LV}, %	0.746	0.925
ET_{LV}, %	0.835	0.975
IRT_{LV}, %	0.757	0.839
FT_{LV}, %	0.885	0.942

RV, right ventricle; LV, left ventricle; P, pulmonary artery valve; A, aortic valve; M, mitral valve; T, tricuspid valve; ICT, isovolumetric contraction time; IRT, isovolumetric relaxation time; ET, ejection time; FT, filling time.
